# Chronic Intestinal Pseudo-Obstruction: Is There a Connection with Gut Microbiota?

**DOI:** 10.3390/microorganisms9122549

**Published:** 2021-12-10

**Authors:** Giulia Radocchia, Bruna Neroni, Massimiliano Marazzato, Elena Capuzzo, Simone Zuccari, Fabrizio Pantanella, Letizia Zenzeri, Melania Evangelisti, Francesca Vassallo, Pasquale Parisi, Giovanni Di Nardo, Serena Schippa

**Affiliations:** 1 Department of Public Health and Infection Disease, Microbiology Section, Sapienza University of Rome, 00185 Rome, Italy; giulia.radocchia@uniroma1.it (G.R.); bruna.neroni@uniroma1.it (B.N.); massimiliano.marazzato@uniroma1.it (M.M.); elis.capuzzo@gmail.com (E.C.); zuccari.simone@icloud.com (S.Z.); fabrizio.pantanella@uniroma1.it (F.P.); 2 Paediatric Unit, NESMOS Department, Faculty of Medicine and Psychology, Sant’Andrea University Hospital, Sapienza University of Rome, 00185 Rome, Italy; zenzeriletizia@gmail.com (L.Z.); melania.eva@gmail.com (M.E.); vassallofrancesca@hotmail.it (F.V.); Pasquale.parisi@uniroma1.it (P.P.); giovanni.dinardo@uniroma1.it (G.D.N.); 3 Paediatric Emergency Department, Santobono-Pausilipon Children’s Hospital, 80129 Naples, Italy

**Keywords:** chronic intestinal pseudo-obstruction, intestinal motility, neurotransmitters, gut microbiota

## Abstract

Chronic intestinal pseudo-obstruction (CIPO) is a rare clinical syndrome characterized by severe impairment of gastrointestinal (GI) motility, and its symptoms are suggestive of partial or complete intestinal obstruction in the absence of any lesion restricting the intestinal lumen. Diagnosis and therapy of CIPO patients still represent a significant challenge for clinicians, despite their efforts to improve diagnostic workup and treatment strategies for this disease. The purpose of this review is to better understand what is currently known about the relationship between CIPO patients and intestinal microbiota, with a focus on the role of the enteric nervous system (ENS) and the intestinal endocrine system (IES) in intestinal motility, underling the importance of further studies to deeply understand the causes of gut motility dysfunction in these patients.

## 1. Introduction

Chronic intestinal pseudo-obstruction (CIPO) disease is a rare, severe gut motility disorder characterized by symptoms of intestinal mechanical obstruction in the absence of detectable anatomical causes. CIPO affects both pediatric and adult patients and it can be primary, secondary or idiopathic in origin. The underlying histopathology may reveal a variety of neuropathy, myopathy, mesenchymopathy or a combination of such abnormalities, e.g., neuro-myopathies. Clinicians do not have unique effective diagnostic tools for CIPO patients, and the most advanced available treatments could only relieve patients’ symptoms. This leads to a late diagnosis and an increased mortality rate for these patients. Motility in the gastrointestinal (GI) tract is controlled by the enteric nervous system (ENS). The ENS acts in concert with enteroendocrine cells (EECs), a large number of cells widely distributed throughout the epithelial lining which secrete bioactive messengers/hormones to regulate various gut functions, including motility, and monitor the endoluminal ecosystem. With over 100 trillion microbial cells in mutualistic association with our intestine, the gut microbiota influences there are the GI physiology, metabolism, nutrition and immune function of the host [1,2,3]. Gut functions are strongly influenced by the gut microbiota, a complex microbial ecosystem that include Bacteria, Archaea, Eucarya and virus which all co-evolved with the host over thousands of years, assembling a complex and mutualistic rapport [4,5,6]. Gut microbiota role, in intestinal motility disorder, should not be neglected. Several studies indicate that gut microbiota interfere with GI motility by different mechanisms, and current discoveries on the inter-relationships between bacteria, viruses and parasites and the ENS are clearly reported in the recent review by Mauro Giuffrè and collegues [7]. In this narrative review, we will discuss current literature regarding the relationship between gut microbiota, CIPO patients and intestinal motility, suggesting that future studies could be important for better understanding the causes of intestinal motility dysfunction in CIPO patients.

## 2. Chronic Intestinal Pseudo-Obstruction (CIPO)

### 2.1. CIPO Etiology and Classification

CIPO is a rare clinical syndrome characterized by impairment of GI motility which resembles mechanical obstruction, in the absence of any obstructive process [8]. The incidence and prevalence of CIPO is still unknown, and the few data available come from small case series [9]. CIPO can affect any segment of the GI tract (the small bowel and the colon are more frequently involved) and represents the most severe form of GI dysmotility [9,10]. CIPO may have different etiologies: primary (familial or sporadic), secondary and idiopathic. Primary CIPO are usually diagnosed in childhood and may be sporadic or familiar [11,12,13]. This condition is characterized by abnormalities within the development, degeneration or inflammation of the ENS and/or of the enteric muscles. Secondary CIPO results from a well-defined disease affecting the intestinal smooth muscle, enteric neurons and the interstitial cell of Cajal (ICC) network and represents up to 50% of the causes in the adult population [14]. The last form of CIPO, defined as idiopathic, is established when neither a primary nor secondary etiology is identified and represents the majority of pediatric CIPO cases [13,15]. Based on abnormal histopathological findings along with the GI system, CIPO has been classified into different groups (neuropathic, myopathic or mesenchymopathic) [11]. Frequently, more than one histopathological alteration coexists in a single CIPO patient.

### 2.2. CIPO Clinical Manifestations

As of histopathological findings and etiologies, CIPO presents a widely variable, non-specific clinical symptomatology [16,17,18,19,20]. It depends on the age at onset, location and extent of the affected GI tract [20,21]. Clinical manifestations start mainly from the first month to the first year of life (80% of the cases) and have a sporadic onset during the first two decades of life (20%) [19,20,21,22,23]. In adult patients, the median age of symptom onset is 17 years [24]. Both pediatric and adult CIPO present unspecific symptomatology such as abdominal pain and distension (80%), vomiting (75%), constipation (40%) and diarrhea (20%) [16,17,19,20,22,23,24,25]. CIPO is characterized by exacerbations and remissions of symptoms during a patient’s life. Exacerbations are triggered by viral or bacterial infections, sepsis, psychological stress or malnutrition. In patients with severe CIPO, the disease could lead to intestinal failure [23]. Moreover, intestinal dilation and slow transit contribute to small intestinal bacterial overgrowth (SIBO), which can cause malabsorption and diarrhea [17]. Malnutrition is a recurrent clinical aspect in CIPO, as ingestion of food generally worsens patients’ symptoms, and the intestinal malabsorption, often associated with dilated bowel loops, alters the regular gut transit [26]. CIPO is also associated with a poor quality of life and high morbidity and mortality [17,27,28].

### 2.3. CIPO Treatments

Regarding CIPO treatment, the use of a single pharmacological agent to treat CIPO patients is rarely effective, and usually several tools are required [29]. CIPO can only be managed with symptomatic therapy aimed to reduce symptom severity, prevent unnecessary surgery, and improve the nutritional status by maintaining an adequate caloric intake, promoting intestinal motility and treating SIBO [30]. Currently, pharmacological therapies used to improve GI dysmotility are prokinetic agents. Erythromycin (macrolide antibiotic), often associated with octreotide, represents a useful option exerting GI prokinetic effects by inducing antrum-duodenal phase III of the migrating motor complex, consequently accelerating the small bowel transit [31,32]. A group of drugs affecting the serotonin (5-hydroxytryptamine or 5-HT) pathway has been tested for CIPO patients’ treatment. In particular, prucalopride is a 5-HT4 receptor agonist with prokinetic properties exerting in accelerating gastric emptying and small-bowel transit [33,34,35]. Other agents useful in improvement GI motility are acetyl-cholinesterase inhibitors (ACIs) (neostigmine and pyridostigmine). Their action in increasing GI motility is well described in children and adults with CIPO refractory to standard therapies [36,37,38,39]. However, to date, these drugs are only able to improve management of some symptoms of CIPO. The second crucial point of CIPO management is to prevent and treat SIBO mainly using antibiotics. Amoxicillin-clavulanate, ciprofloxacin, doxycycline and metronidazole are the agents most used to improve abdominal distention and pain in CIPO patients [40]. Nevertheless, the most recent recommended agent is rifaximin, a poorly absorbed antibiotic that, differently to the others, exerts non-traditional effects on the gut microbiota in addition to bactericidal/bacteriostatic activity, producing lower bacterial resistance than traditional agents [41,42]. Its administration improves SIBO-associated symptoms and breath test results [43,44]. Finally, fecal microbiota transplantation (FMT) has been recently proposed as a new therapeutic option for CIPO patients. A pilot study, conducted by Gu and collaborators, demonstrated that FMT significantly improves patients’ conditions, alleviating pain and bloating symptoms and eliminating SIBO in 71% of patients after only two weeks of treatment [45].

## 3. Gastrointestinal (GI) Motility, Enteric Nervous System (ENS) and Intestinal Endocrine System (IES)

### 3.1. GI Motility

Intestinal motility is a complex function coordinated by the central nervous system (CNS) that involves an interaction between the ENS, the smooth muscle cell contractile system, the ICC and the afferent and efferent nerve fibers. ENS comprises approximately 600 million neurons embedded within smooth muscle of the GI tract, organized in microcircuits, with interneurons and intrinsic primary afferent neurons (IPANs), which are capable of triggering reflexes. There is a strict bidirectional communication between the CNS and the ENS: brain influences enteric behavior and vice versa [46]. Signals received in the brain allow neurons within the CNS to respond to enteric stimuli for the regulation of motility patterns in the esophagus and stomach [46,47]. The ENS controls movements of the small and large bowel. The ENS integrity is essential: interruption or damage to any of the machineries in GI motility can result in an intestinal motility disorder.

### 3.2. ENS, IES and Neurotransmitters

ENS acts in concert with the intestinal endocrine system (IES). IES, located in the GI tract, consists of EECs dispersed along the whole intestinal mucosa in the villi and crypts. The EECs secrete bioactive messengers/hormones to regulate various gut functions, including motility, and to monitor the endoluminal ecosystem [48]. The ENS controls GI motility through neurons interconnected by neurotransmitters, such as dopamine, acetylcholine and 5-HT. Dopamine is a precursor for other catecholamines, such as norepinephrine and epinephrine. These have different roles in sensory signal detection, behavior and conditions such as memory, attention and learning [49]. Acetylcholine is the main neurotransmitter of peripheral nerve fibers, and its main functions are: stimulate contraction of smooth muscles, increase bodily secretions, dilate blood vessels and slow heart rate. 5-HT regulates different physiological processes, such as GI secretion and peristalsis, vasoconstriction, behavior, respiration and neuronal functions [50,51]. The efficacy of treatments with drugs that affected acetylcholine, 5-HT and GABA mediator systems in CIPO patients confirm the importance of neurotransmitters in CIPO pathology. For instance, cisapride, a 5-HT4 receptor agonist and a 5-HT3 antagonist with prokinetic properties, binds to the 5-HT receptors in the myenteric plexus, inducing an acetylcholine release and the smooth muscle contractions, resulting in an increase of post-prandial duodenal contractions in CIPO patients [33,34]. Moreover, prucalopride, another 5-HT4 receptor agonist, facilitates acetylcholine release, resulting in the activation of cholinergic neurotransmission, accelerating gastric, small-bowel and colonic transit [35]. It is particularly important to clarify how gut microbiota may modulate gene expression, synthesis and/or function of these neurotransmitters.

## 4. The 5-Hydroxytryptamine (5-HT), Serotonin

A subset of EECs, the enterochromaffin cells (EC), upon mechanical, chemical or neural stimulation releases the amine 5-hydroxytryptamine which acts binding to specific receptors (e.g., 5-HT4), thereby eliciting peristaltic reflexes and propulsive motility [52,53,54,55,56,57]. Most of the endogenous 5-HT is synthesized within the EECs in the GI mucosa via the enzyme tryptophan hydroxylase-1 (Tph1) [58]; a minimal amount of 5-HT is synthesized in the ENS via the enzyme Tph-2 [59,60]. 5-HT is a pleiotropic amine that has long been assumed to play an important role in several gut functions, since it has been initially isolated and localized into EECs by Erspamer in 1937 [48]. It has been demonstrated that 5-HT biosynthesis is modulated by different factors: variations in luminal glucose levels [61], increases in luminal short-chain fatty acids (SCFAs) derived from bacteria [62], neuromodulators agents derived from the CNS and/or the ENS [60,63,64,65,66]. The stimulating or inhibiting effect of 5-HT in the different parts of the organism is related to the site and type of serotonergic receptor involved. The membrane receptors are present both in the CNS and in the peripheral one, as well as in non-neuronal tissues such as blood or GI, endocrine, sensory, cardiovascular and other systems [67]. The most recent classification of 5-HT receptors (serotonergic), proposed by IUPHAR in 1998 and still current, suggests the subdivision on the basis of pharmacological and structural characteristics into seven classes: 5-HT1, 5-HT2, 5- HT3, 5-HT4, 5-HT5, 5-HT6 and 5-HT7. Among these, the 5-HT3 and the 5-HT4 are mainly expressed in the GI tract [68,69,70]. These two receptors, expressed by functionally distinct enteric neurons, the smooth muscle cells and the secretory cells, are able to promote peristalsis and evoke cholecystokinin secretion [71,72]. The reuptake of 5-HT into epithelial cells is necessary to deactivate its action, whereas accumulation of 5-HT in the interstitial cleft may cause receptor desensitization [73]. Only recently a specific 5-HT transporter (SERT), involved in 5-HT reuptake, expressed in mucosal epithelial cells of several animals and humans, has been detected [69,70,74,75,76,77]. This is a new variant of specific SERT, in addition to the one expressed by the nervous system [73,78,79]. Alterations in mucosal 5-HT levels linked to variations in the expression of *Tph-1* and *SERT* genes, or in the expression of 5-HT intestinal receptors genes *5-HT3* and the *5-HT4*, has been reported in intestinal biopsies of patients with Irritable Bowel Syndrome (IBS) [80]. Based on the success of treatments with drug antagonists of the intestinal receptors of 5-HT, it could be assumed that in CIPO patients, intestinal dysmotility could be linked to a serotonergic pathway malfunction.

## 5. Gut Microbiota Interplay with CIPO Patients

The GI tract represents the largest surface colonizable in the human body. The microbes’ community is collectively referred to as gut microbiota and strongly impacts host homeostasis and disease. Gut microbiota is considered a second genome that actively modulates human health [81]. With over 100 trillion of microbial cells in mutualistic association with our intestine, the gut microbiota influences the GI physiology, metabolism, nutrition and immune function of the host [82,83,84]. A condition in which bacteria no longer live in a mutualistic relationship is defined as dysbiosis. Currently, dysbiosis has been linked with important human diseases. Microbiota composition, diversity and metabolic activity resulted in being altered in several GI disorders, such as inflammatory bowel disease (IBD) [83], celiac disease [85], IBS [86] and obesity [85]. CIPO exacerbations can be triggered by viral or bacterial infections, psychological stress or malnutrition, all factors influenced by gut microbiota composition [87]. Viral and bacterial infections could have a negative impact on gut microbiota and clinical diseases [88]. The most common infectious cause for secondary CIPO is Chagas’ disease, caused by the protozoan *Trypanosoma cruzi* [4]. In addition, it is known that intestinal dilation and slow transit contribute to SIBO, defined as an excessive presence of bacteria in the small intestine, often reported in CIPO patients. An altered gut microbiota, which could lead to intestinal epithelial barrier dysfunction and immune dysregulation, could also affect intestinal neuromuscular homeostasis, representing a risk factor that triggers a severe gut dysmotility [17]. Considering the potential important role of microbiota in CIPO, Gu and collaborators conducted an experimental FMT treatment in nine CIPO patients [45]. FMT is a new diagnostic tool, already used in conditions associated with severe dysbiosis, which aims to restore gut microbiota by the administration of fecal material from a healthy subject to a dysbiotic patients [5]. After 8 weeks from the FMT, a relief of symptoms for pain and bloating was observed in CIPO patients. These results suggested that FMT might be beneficial also for the treatment of CIPO, but further studies are required to confirm these findings [45]. Finally, our group is currently studying the composition of mucosa-associated microbiota in pediatric CIPO patients. Our preliminary results (data not yet published) highlighted a specific composition and biodiversity of gut microbiota in these patients with respect to pediatric controls with negative diagnosis of CIPO. This brief overview suggests a possible microbiota involvement in the pathogenesis of CIPO. In Table 1, several studies correlating gut microbiota and CIPO are summarized.

## 6. Gut Microbiota Interplay with Gastrointestinal Motility

The ENS controls GI motility where neurons are interconnected through neurotransmitters, counting dopamine, acetylcholine and serotonin. The gut appears to affect the enteric and the CNS development and disorders, such as neurodegenerative diseases, cerebrovascular accidents and behavioral, neuroimmune-mediated or motility disorders. The interaction pathways along the “gut−brain axis” encompass those determined by the immune system, the vagus nerve or the variation of neuroactive microbiota compounds [89]. Gut bacteria are able to influence an extensive variety of mammalian neurotransmitters, including serotonin, norepinephrine, dopamine or gamma-aminobutyric acid (GABA). The bacteria’s impact on these neurotransmitters could have an influence on the host’s health status. Several bacteria have been described as being able to produce neurotransmitters [1]. Studies carried out on animal models or humans displayed that interventions centered on microbiota can modify the levels of neurotransmitters [89]. Two recent studies showed that germ-free animals have significant alterations in several ENS/CNS-related functions, including intestinal motility [2,3].

### 6.1. Bacteria’s Influence on Serotonin

Among neurotransmitters, serotonin regulates several physiological processes, counting peristalsis, GI excretion, vasoconstriction, respiration, neurological function and behavior [50,51]. A recent study shows that the gut microbiota modulates the functionality and anatomy of the ENS through the induction of serotonin release and the activation of its 5-HT4 receptor [90]. An additional recent study, conducted on a mouse model, investigated the link between gut microbiota and the protein SERT [91], showing that *SERT* gene deletion is linked with gut dysbiosis. It has been demonstrated that serotonin biosynthesis is modulated by different factors: variations in luminal glucose levels [61], increasing in luminal SCFAs derived from bacteria [62], neuromodulator agents derived from the CNS and/or the ENS [92,93,94]. A healthy gut microbiota provides signals to host mucosal EC cells to maintain gut serotonin content, stimulating Tph-1 expression by specific microbiota metabolites, such as SCFAs or secondary bile acids [3,62]. Germ-free animals showed a significant serotonin decrease in blood and colon districts compared to colonized controls [3,95].

### 6.2. Bacteria’s Influence on GABA

GABA is an inhibitory neurotransmitter of the CNS and, together with its receptor, is extensively disseminated through the mammalian host. Altered GABA level is linked with plentiful CNS disorders, counting behavioral disorders, pain, sleep [96] and also interference with ENS functions, such as gastric emptying, intestinal motility and acid secretion [97]. A study conducted in germ-free animals showed a reduction in serum and luminal levels of GABA (not cerebral) [98]. Some microorganisms are GABA producers, in particular the genera Bifidobacterium and Lactobacillus. Among those species, *Lactobacillus rhamnosus JB-1* was found able to diminish depressive and anxiety-like behavior when introduced into mice [99]. These collected results suggest that gut microbiota has influences on the nervous system, but further studies are mandatory to clarify these mechanisms.

### 6.3. Bacteria’s Influence on Norepinephrine or Dopamine

Dopamine is a neurotransmitter affecting behavior and a precursor of norepinephrine, epinephrine and other catecholamines. Norepinephrine has a role in stimulation and vigilance of the wakening state and in revealing the sensorial signal; besides, it seems also to be involved in behavior and cognition, such as memory, learning and attention [49]. It is thought that bacteria respond to catecholamines and are able to produce them. *Escherichia coli O157:H7* (EHEC) increases its growth rate when dopamine and norepinephrine are present [100], and in the presence of norepinephrine displays an increase in motility, biofilm development and virulence [101]. Furthermore, *Klebsiella pneumoniae*, *Pseudomonas aeruginosa*, *Enterobacter cloacae*, *Shigella sonnei* and *Staphylococcus aureus* showed an enhanced growth in the presence of norepinephrine, probably due to iron acquisition [102]. Numerous bacteria are also able to produce dopamine and norepinephrine in vitro: *Bacillus subtilis*, *Serratia marcescens*, *E. coli* and *Proteus vulgaris* [103]. It seems that norepinephrine is a quorum sensing molecule in bacteria; otherwise, dopamine production is not yet understood [104]. Different studies support that microbiota could modulate norepinephrine or dopamine in vivo or that they could play a role in host biosynthesis/catabolism, but this has not been confirmed yet [89]. Germ-free mice have decreased levels of norepinephrine in cecal lumen and tissue, and norepinephrine cecal levels might be reestablished via microbiota colonization or with a combination of 46 Clostridia species [1]. Results suggest that microbiota affects lumen norepinephrine levels, but do not confirm bacteria norepinephrine production. In microbiota-depleted mice, an improved sensitivity to the behavioral effects of cocaine has been reported, and the social response to cocaine was regularized with SCAFs, indicating an indirect impact of microbiota influencing their behavior [105].

Several studies seems to corroborate the idea that gut bacteria impact a wide variety of neurotransmitters, including those involved in the GI motility [1,2,3,7,89,90,91]. In Table 2, several studies showing the impact of gut microbiota on neurotransmitters are summarized. Moreover, our preliminary results showed significant differences in the expression levels of genes related to serotonin intestinal synthesis and its reuptake in CIPO patients with respect to controls (data not yet published).

## 7. Conclusions

The composition of gut microbiota plays an important role in intestinal motility disorder pathogenesis. Although the knowledge on the role gut microbiota in GI function, including GI motility, is improved, there are limited data on the connections between gut microbiota, ENS, IES and ECCs in CIPO patients. Abnormalities in neurotransmitter signaling pathways, which could be triggered/linked by alteration of gut microbiota or its products, could underlie intestinal motility dysfunction in CIPO patients. Studies aiming to evaluate any possible correlations between gut microbiota and factors related to intestinal motility could help clinicians to better understand the CIPO pathogenesis, highlight CIPO microbial biomarkers, as well as new therapeutic targets, improving the management and treatment of these patients.

## Figures and Tables

**Table 1:** Gut microbiota and CIPO patients.

Author of the Study and Year of Publication	Results	Reference
Stanghellini et al., 2005	Intestinal dilation and slow transit contribute to SIBO	[17]
Gu et al., 2017	FMT treatment improves symptoms of pain and bloating	[45]
Stanghellini et al., 2005	An altered gut microbiota leads to intestinal epithelial barrier dysfunction and immune dysregulation, representing a risk factor that triggers a severe gut dysmotility	[17]
Karl et al., 2018	CIPO exacerbations can be triggered by viral or bacterial infections	[87]
Rodriguez dos Santos et al., 2018	The protozoan *Trypanosoma cruzi* causes Chagas’ disease in secondary CIPO	[4]

**Table 2:** Impact of gut microbiota on neurotransmitters.

Author of the Study and Year of Publication	Results	Reference
Asano et al., 2012	Bacteria are able to produce neurotransmitters	[1]
Strandwitz et al., 2018	In human and mouse models, interventions centered on microbiota composition modify the levels of neurotransmitters	[89]
Dey et al., 2015; Yano et al., 2015	Germ-free animals show alterations in different ENS- and CNS-related functions	[2,3]
Gershon et al., 2007; Berger et al., 2009	Serotonin regulates several physiological processes, e.g., peristalsis	[50,51]
De Vadder et al., 2018	Gut microbiota modulates the functionality and anatomy of the ENS through the serotonin release	[90]
Reigstad et al., 2015	Serotonin biosynthesis is modulated, among other factors, by luminal SCFAs derived from bacteria	[62]
Yano et al., 2015; Reigstad et al., 2015	Specific metabolites of gut microbiota provide signals to host mucosal EC cells, stimulating *Tph-1* expression	[3,62]
Yano et al., 2015; Wikoff et al., 2009	In germ-free animals, serotonin levels decrease in blood and colon districts	[3,95]
Hyland et al., 2010	GABA levels interfere with ENS functions, such as intestinal motility	[97]
Matsumoto et al., 2013	Germ-free animals show a reduction in serum and luminal levels of GABA (not cerebral)	[98]
Bravo et al., 2011	Specific microorganisms produce GABA	[99]
Strandwitz et al., 2018	Microbiota can modulate norepinephrine or dopamine in vivo	[89]
Asano et al., 2012	Germ-free mice have decreased levels of norepinephrine in cecal lumen, which could be reestablished via microbiota colonization	[1]
